# EORTC-SPECTA Arcagen study, comprehensive genomic profiling and treatment adaptation of rare thoracic cancers

**DOI:** 10.1038/s41698-024-00518-9

**Published:** 2024-02-16

**Authors:** Marco Tagliamento, Marie Morfouace, Charalambos Loizides, Julio Oliveira, Laurent Greillier, Judith Raimbourg, Anne-Claire Toffart, Thierry Chatellier, Nicolas Cloarec, Ivana Sullivan, Birute Brasiuniene, Michael Duruisseaux, Kersti Oselin, Marie-Sophie Robert, Carolina Fernandes, Arnaud Poncin, Jean-Yves Blay, Benjamin Besse, Nicolas Girard

**Affiliations:** 1 Department of Cancer Medicine, Gustave Roussy, Villejuif, France; 2 Department of Internal Medicine and Medical Specialties, University of Genova, Genova, Italy; 3 EORTC Headquarters, Bruxelles, Belgium; 4 Medical Oncology Department, Instituto Portugues de Oncologia do Porto Francisco Gentil, Porto, Portugal; 5 Aix Marseille University, APHM, INSERM, CNRS, CRCM, Hôpital Nord, Multidisciplinary Oncology and Therapeutic Innovations Department, Marseille, France; 6 Department of Medical Oncology, Nantes Université, Institut de Cancerologie de l’Ouest, Saint-Herblain, France; 7 Grenoble Alpes University, Grenoble Alpes Hospital, Grenoble, France; 8 Clinique Mutualiste de l’Estuaire – Centre d’Oncologie, Saint Nazaire, France; 9 Service d’Oncologie Médicale et Hématologie Clinique, Centre Hospitalier d’Avignon, Avignon, France; 10 Medical Oncology Department, Hospital de la Santa Creu i Sant Pau, Barcelona, Spain; 11 Department of Medical Oncology of National Cancer Institute, Faculty of Medicine of Vilnius University, Vilnius, Lithuania; 12 Department of Medical Oncology, CHU de Lyon - Hôpital Lyon Sud, Lyon, France; 13 Department of Chemotherapy, Clinic of Oncology and Hematology, North Estonia Medical Centre, Tallinn, Estonia; 14 Department of Medicine, Centre Léon Bérard, Lyon, France; 15 Paris-Saclay University, Department of Cancer Medicine, Gustave Roussy, Villejuif, France; 16 Institut du Thorax Curie Montsouris, Institut Curie, Paris, France

**Keywords:** Molecular medicine, Cancer genomics, Predictive markers

## Abstract

Arcagen (NCT02834884) is a European prospective study aiming at defining the molecular landscape of rare cancers for treatment guidance. We present data from the cohort of rare thoracic tumors. Patients with advanced pleural mesothelioma (PM) or thymic epithelial tumors (TET) underwent genomic profiling with large targeted assay [>300 genes, tumor mutational burden (TMB), microsatellite instability (MSI) status] on formalin-fixed paraffin-embedded (FFPE) or plasma samples. EORTC molecular tumor board (MTB) advised for biomarker-guided treatments. 102 patients recruited from 8 countries between July 2019 and May 2022 were evaluable: 56 with PM, 46 with TET (23 thymomas, 23 thymic carcinomas). Molecular profiling was performed on 70 FFPE samples (42 PM, 28 TET), and 32 cases on ctDNA (14 PM, 18 TET), within a median turnaround time of 8 days from sample reception. We detected relevant molecular alterations in 66 out of 102 patients (65%; 79% PM, 48% TET), 51 of 70 FFPE samples (73%; 90% PM, 46% TET), and 15 of 32 plasma samples (47%; 43% PM, 50% TET). The most frequently altered genes were *CDKN2A/B, BAP1, MTAP* in PM and *TP53, CDKN2A/B, SETD2* in TET. The TMB was low (mean 3.2 Muts/MB), 2 PM had MSI-high status. MTB advised molecular-guided treatment options in 32 situations, for 17 PM and 15 TET patients (75% clinical trial option, 22% off-label drug or compassionate use, 3% early access program). Molecular testing and MTB discussion were feasible for patients with rare thoracic cancers and allowed the broadening of treatment options for 30% of the cases.

Pleural mesothelioma (PM) and thymic epithelial tumors (TET) are rare aggressive thoracic malignancies. There are 30,000 new PM cases per year worldwide, with an incidence in Europe of <1 per 100,000 per year^1^. It is related to asbestos exposure, with clinical onset occurring after decades of latency. Histological classification into three major subtypes - epithelioid, sarcomatoid, and biphasic - is important in terms of prognosis and treatment^2^. The most recent therapeutic regimens consist of combination with immunotherapy targeting PD-(L)1 and CTLA-4, and antiangiogenics, bringing the median overall survival of patients with unresectable PM up to 1.5 years^3–5^. However, when progressing, there is a lack of effective treatment options. The annual incidence of TET (thymomas and thymic carcinomas together) is 1.3–3.2 cases per million worldwide^6^. Thymomas are subdivided into sub-types, and tumor stage and resection status are fundamental prognostic factors^7^. Multimodal treatments including radiotherapy, surgery and chemotherapy are key to disease control. Multi-tyrosine kinase inhibitors, biological agents and immunotherapy may have a role in the metastatic recurrent setting^2^, although effective predictive biomarkers are lacking^8^. The low incidence of these diseases hindered the running of randomized trials for new therapeutic strategies. Rare cancers are also under-represented in genomic profiling programs. All this contributes to the scarcity of targeted therapies available for PM and TET^2^.

Arcagen is a European Organisation for Research and Treatment of Cancer (EORTC)-SPECTA prospective multicohort and multicenter study aiming at performing genomic profiling, associated with a molecular tumor board (MTB) for treatment recommendation, for patients with rare cancers across Europe. Here we present the first results of the cohort of rare thoracic malignancies.

## Characteristics of the study cohort

A total of 122 patients from 8 different countries were registered between July 2019 and May 2022 (Supplementary Figure 1 and Supplementary Table 1). 102 patients (84%) were evaluable, with adequate samples for molecular profiling: 56 PM and 46 TET (23 thymic carcinomas and 23 thymomas). Patient and disease characteristics are summarized in Table 1. At study enrollment, PM patients were older than TET (72 and 60 years, respectively). There was a prevalence of men among PM patients (79%) and of women in the TET cohort (54.3%). 58% of the patients had been exposed to tobacco (61% PM, 54% TET). All histologic subtypes of MP and thymomas were represented. Most of the patients were pretreated in the advanced setting at the time of the MTB discussion (up to 5 previous lines of therapy) (Table 1).

**Table 1:** Patient and disease characteristics.

	All cancers, *n* = 102 *N* (%)	Mesothelioma, *n* = 56 *N* (%)	Thymic epithelial tumors, *n* = 46 *N* (%)
**Age, median [IQR]**	70 [55–74]	72 [67–75]	60 [43–70]
**Sex, female**	37 (36%)	12 (21%)	25 (54%)
**Smoking status**			
Never	39 (38%)	21 (38%)	18 (39%)
Former	48 (47%)	29 (52%)	19 (41%)
Current	11 (11%)	5 (9%)	6 (13%)
NA	4 (4%)	1 (2%)	3 (7%)
			Thymic carcinoma: 23 (50%)
			Thymoma: 23 (50%)
**Histology**		• Epithelioid: 33 (59%) • Sarcomatoid: 4 (7%) • Biphasic: 5 (9%) • Others: 4 (7%) • NA: 10 (18%)	• A: 1 (4%) • AB: 1 (4%) • B1: 1 (4%) • B1/B2: 1 (4%) • B2: 6 (26%) • B2/B3: 4 (17%) • B3: 4 (17%) • NA: 5 (22%)
**Localized or locally advanced disease at diagnosis**	29 (28%)	5 (9%)	25 (54%)
**Number of previous lines of therapy for metastatic disease, median (range)**	1 (0–5)	1 (0–5)	1 (0–5)
**Biopsy site/specimen**			
Primitive tumor	48 (47%)	35 (62%)	12 (26%)
Nodal metastasis	2 (2%)	2 (4%)	—
Metastasis other	20 (20%)	5 (9%)	16 (35%)
Blood	32 (31%)	14 (25%)	18 (39%)
**MSI-status**			
High	2 (2%)	2 (4%)	—
Stable	88 (86%)	51 (91%)	37 (80%)
NA	12 (12%)	3 (5%)	9 (20%)
**TMB Muts/MB, mean (range)**	3.6 (0–61)	4 (0–61)	3.1 (0–15)
	NA: 13	NA: 3	NA: 10

*IQR* interquartile range, *MB* megabase, *Muts* mutations, *MSI* microsatellite instability, *NA* not available, *TMB* tumor mutational burden.

## Samples analysis

**Fig. 1:**
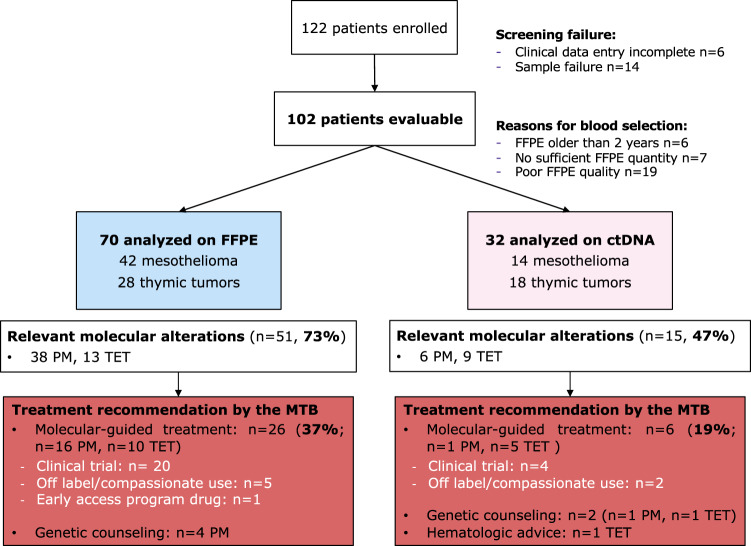
Sample workflow and MTB recommendations. Workflow summarizing the operational settings of the EORTC Arcagen project from patient enrollment to molecular tumor board recommendations. In pink, molecular analysis performed on plasma, in blue on FFPE samples.

Figure 1 describes the sample workflow and reasons for rescue with liquid biopsy. Considering all tumor types together, the molecular analysis of PM was performed on formalin-fixed paraffin embedded (FFPE) tissue samples in 42 cases (75%), while 14 cases (25%) were analyzed by liquid biopsy. Conversely, the genomic analysis was done on tissue in 28 TET patients (61%), while 18 cases were tested by liquid biopsy (39%). The main reason for rescue was poor FFPE quality (19 cases, 60%), no sufficient FFPE available (22%) or FFPE material older than 2 years (18%). The median turnaround time (TAT) from samples acquisition from each center to the molecular report was 8 days for both analyses (thus excluding the previous steps of patient registration and sample preparation).

## Genomic profiling results and MTB recommendations

**Fig. 2:**
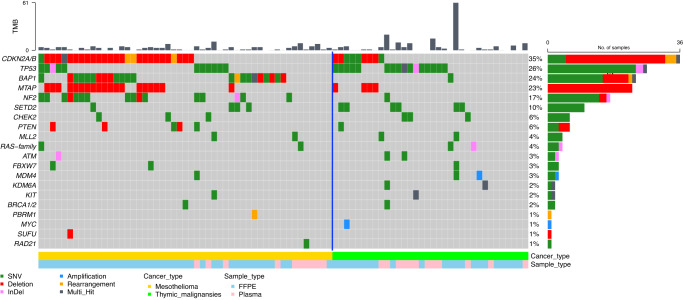
Oncoplot of the 20 most common molecular alterations. Oncoplot summarizing the main molecular alterations in mesothelioma (yellow squares, left part of the plot) and thymic malignancies (green squares, right part of the plot). TMB is highlighted at the top of the figure. Molecular alterations: SNV in green, deletion in red, InDel in pink, amplification in blue, rearrangement in yellow and multi-hits in dark grey.

The oncoplot in Fig. 2 reports the 20 most frequently detected alterations in each cancer type. We detected relevant molecular alterations in 66 out of 102 patients (65%). To note, no ESCAT tier 1 or 2 molecular targets were detected. MTB recommendations are summarized in Fig. 1.

### Pleural mesothelioma

Overall, 79% of PM cases (*n* = 44) harbored at least one molecular alteration. The most frequent aberrations in MP were deletions or rearrangements of *CDKN2A/B*, single nucleotide variants (SNV) or deletions of *BAP1* and *NF2*, deletions of *MTAP* and SNV of *TP53* (Supplementary Fig. 2). The detection rate was higher in FFPE, with alterations identified in 38 out of 42 samples (90%), while it was limited to 6 out of 14 plasma samples (43%). The tumor mutational burden (TMB) was generally low (mean 4 Muts/MB), however 2 PM cases had microsatellite instability (MSI)-high status. MTB advised for potential treatment options in 17 situations (30% of the PM cohort). Recommendation for germline testing and eventual genetic counseling to be performed locally was given for 5 patients, mostly due to the detection of pathogenic variants in *BAP1* of suspected germline origin.

### Thymic epithelial tumors

In TET, 48% of the cases (*n* = 22) carried at least a molecular alteration. We mostly detected SNV of *TP53*, SNV or deletions of *CDKN2A/B*, SNV of *SETD2* and deletions of *MTAP* (Supplementary Fig. 3). For TET, the detection rate was 50% in plasma samples (9 out of 18 cases) and 46% in FFPE samples (13 out of 28 cases). The TMB was generally low (mean 3.1 Muts/MB). MTB advised for potential treatment options in 15 situations (33%).

One hematologic consultation was suggested due the detection of clonal hematopoiesis in liquid biopsy, associated with an increased risk of developing a hematologic neoplasm.

### Combined analysis

Considering PM and TET together, the detection rate of pathogenic or likely pathogenic molecular alterations was higher in FFPE samples than in liquid biopsy (73% vs 47%). The MTB advised for treatment options in 32 situations (75% clinical trial option, 22% off label drug or compassionate use, 3% early access program) (Supplementary Fig. 4). Hence, a molecular-matched treatment indication as further line of treatment in case of disease progression was given for 31% of the overall population. Overall, tissue and plasma analysis informed the treatment indication in 37% and 19% of the cases, respectively. A detail of MTB treatment indications is reported in Supplementary Table 2. For 11 patients there was no treatment adaptation based on the MTB recommendation due to an early death for disease progression, and in 19 cases the disease had not progressed at time of database lock.

Comprehensive genomic profiling has emerged as a powerful tool for understanding the biology of rare cancers and identifying potential targets for therapy. Overall, we show that genomic profiling has the potential to inform personalized treatment strategies for 31% of patients with advanced rare thoracic tumors in an efficient manner (TAT of 8 days from receipt of the sample), with treatment recommendation discussed during a pan-Europe MTB. The low failure rate (less than 20%) and successful operational set-up of the project highlight the feasibility of systematic screening for rare thoracic cancers.

Previous studies have explored the molecular landscape of PM and TET using a variety of genomic profiling techniques. Results of our analysis are in line with literature reports on PM^9–13^, which have identified a number of recurrent genetic alterations and signaling pathways that may contribute to the development and progression of PM. Some of the key findings include frequent mutations in tumor suppressor genes such as *CDKN2A/B, TP53, BAP1*, and *NF2*, as well as in genes involved in chromatin remodeling, such as *SETD2*. Mutations in DNA damage response genes were also represented. We confirmed that also TET exhibit a diverse range of genomic alterations involving cell cycle and chromatin regulation^8,14^. Most frequently altered genes include *TP53 (*one Y220C mutant, target of future potential drug)^15^, loss of *CDKN2A* and *MTAP*, mutations in *KIT*, *PTEN* and *RAS* family genes. Nevertheless, while genomic profiling has provided valuable insights into the molecular landscape of these cancers, previous studies did not address how this may translate into clinical utility. Indeed, many unanswered questions and major barriers exist on this regard, such as the definition of the clinical relevance of detected molecular alterations, clinical trials availability at the time of tumor profiling and drugs accessibility. The aim of our study was to bypass these limitations, by providing an efficient and clinically relevant molecular profile in support to patients and treating clinicians for improving the management of rare thoracic cancers in Europe. This is also key to better select patients for clinical trials assessing targeted therapies, considering that the 75% of the MTB recommendations was a clinical trial option (phase I or basket trial in most of the cases).

Limitations to this study include a short follow-up after enrollment, limiting the availability of data about the implementation of MTB recommendations, thus possible clinical benefit associated with it. Nevertheless, we noticed that in 34% of cases with a MTB recommendation, death due to disease progression hampered the possibility to adapt the therapy, suggesting that tumor profiling should be performed earlier at cancer diagnosis rather than during treatment for metastatic disease, when clinical conditions could prevent the inclusion in a clinical trial. Another pitfall relies into the intrinsic limitation of using circulating tumor DNA (ctDNA) for tumor genotyping. The detection rate may have been overestimated, particularly in the liquid biopsy cohort, due a misinterpretation of some clonal hematopoiesis variants (e.g., *TP53*, that is non univocally somatic). Nevertheless, previous evidence showed 80% detection of at least one molecular alteration through ctDNA analysis in TET patients^16^, and mutations and rearrangements could be preferentially detected in liquid than tissue biopsy^17^. The inconsistent detection rate in PM and TET could be largely explained by clinical-pathological differences influencing ctDNA shedding^18^.

Overall, the results of this study highlight the role of academic MTB in expanding diagnostic and targeted treatment options for patients affected with rare thoracic tumors and demonstrate the operational feasibility on a multinational scale. Tumor tissue sequencing remains the best approach for molecular characterization, but liquid biopsy may play an important role in cases with inadequate tissue samples. Access to drugs remains critical for patients with rare cancers.

## Methods

Patients older than 12 with a diagnosis of locally advanced or metastatic mesothelioma of pleura and pericardium, thymoma, thymic carcinoma, regardless of the number of treatment lines received, were recruited. A recent tumor specimen was requested for analysis (less than 2-year-old). If non available, or failed quality control at the central biobank, a liquid biopsy was performed. Clinical-pathological data were collected according to the EORTC-SPECTA protocol (NCT02834884)^19^. All patients provided written informed consent for sample collection and genomic analysis. The SPECTA Arcagen study was approved by ethical committees—Commissie voor Medische Ethiek ZNA (Belgium), reference 4881, date 10/03/2021—Comissao de Etica para a Saude de CHLO (Portugal), reference 2150, date 28/06/2021—Eticka komise pro Multicentricka Klinicka Hodnoceni, Fakultini Nemocnice v Motole (Check Republic), reference 017/08, date 01/12/2021. This study has been conducted in accordance with international standards of ethical principles for medical research (Declaration of Helsinki, Good Clinical Practice guidelines).

Comprehensive genomic profiling was performed with a targeted next generation sequencing (NGS) test (FoundationOne®CDx) of >300 genes, including MSI and TMB, using DNA isolated from FFPE tumor tissue specimens. If tumor tissue was not available or inadequate, the analysis was performed by FoundationOne®Liquid CDx on ctDNA isolated from plasma. FoundationOne®CDx and FoundationOne®Liquid CDx have high accuracy and analytical sensitivity, although there may be some differences between the two tests due to the nature of the sample they analyze. Results also depend on the specific genetic alterations being analyzed, tumor heterogeneity, and the quality of the sample. No TMB calculation was reported at the beginning of the project using FoundationOne®Liquid CDx.

Each individual genomic report was reviewed and discussed by the EORTC-SPECTA multidisciplinary MTB, which met monthly, composed of experienced oncologists, biologists, bioinformatics and geneticists, expert in molecular medicine and thoracic cancers management. Its primary objective was to analyze the NGS report to suggest personalized treatment strategies in the context of the patient pathology and status. This process involved evaluating, in addition to what was concluded in the NGS report, the pathogenicity and the targetability of individual molecular alterations according to the ESMO Scale for Clinical Actionability of molecular Targets (ESCAT). Only pathogenic or likely pathogenic variants were considered. Matched therapies and targeted agents were recommended where possible based on existing preclinical and clinical data, available within clinical trials not restricted to EORTC trials, or outside a trial.

### Reporting summary

Further information on research design is available in the Nature Research Reporting Summary linked to this article.

### Supplementary information

REPORTING SUMMARY

Supplementary Information file

## Data Availability

Data can be access in line with EORTC data sharing policy (https://www.eortc.org/app/uploads/2023/06/L-01-POL-01.pdf). The link for data request can be found here: https://www.eortc.be/services/forms/erp/request.aspx. Sequencing data generated in this study were uploaded in the European Genome-phenome Archive (EGA), dataset reference number EGAD50000000168.
