# Supplementary material for: EORTC-SPECTA Arcagen study, comprehensive genomic profiling and treatment adaptation of rare thoracic cancers
Source: NPJ Precis Oncol. 2024 Feb 16;8:37. doi: 10.1038/s41698-024-00518-9 (PMC10873296; doi:10.1038/s41698-024-00518-9)
Supplement: Supplementary file 2 — Supplementary Information file [file 41698_2024_518_MOESM2_ESM.pdf]

SUPPLEMENTARY MATERIAL

Supplementary Table 1: List of participating centers

Center name	Country	Registered	Enrolled
Cen.Hospitalar Vila Nova Gaia	PT	2	1
Hospital Universitario Infanta Leonor	ES	4	0
U.Z. Leuven - Campus Gasthuisberg	BE	2	2
CUF hospitals - Hospital CUF Tejo	PT	2	2
C.H.U. Sart-Tilman	BE	5	5
CHU de Lyon - Hopital Lyon Sud	FR	3	3
North Estonia Medical Centre - Mustamäe unit	EE	3	2
CHU de Grenoble - La Tronche - Hopital A. Michallon	FR	11	8
Centre Hospitalier Avignon	FR	7	7
Gustave Roussy	FR	11	9
Centre Oscar Lambret	FR	1	1
Institut de Cancerologie de l'Ouest (ICO) - Institut de Cancerologie de l'Ouest (ICO) - Centre Rene Gauducheau	FR	10	9
Centre Eugene Marquis	FR	1	0
Assistance Publique - Hopitaux de Marseille - Hopital Nord (APHM)	FR	14	13
Academisch Ziekenhuis Maastricht	NL	2	2
Hospital Clinic Universitari de Barcelona	ES	6	3
Hospital De La Santa Creu I Sant Pau	ES	6	4
Lithuanian Oncology Center-Nat. Cancer Inst	LT	3	3
Institut du Cancer de Montpellier	FR	3	2
The Christie NHS Foundation Trust	UK	1	1
Cambridge University Hospital NHS - Addenbrookes Hospital	UK	1	0
Instituto Portugues De Oncologia - Instituto Portugues de Oncologia do Porto	PT	19	17
Clinique Mutualiste de l'Estuaire - Centre d'oncologie	FR	7	7
CUF hospitals - Hospital CUF Descobertas	PT	1	0

Supplementary Figure 1: Map of participating countries

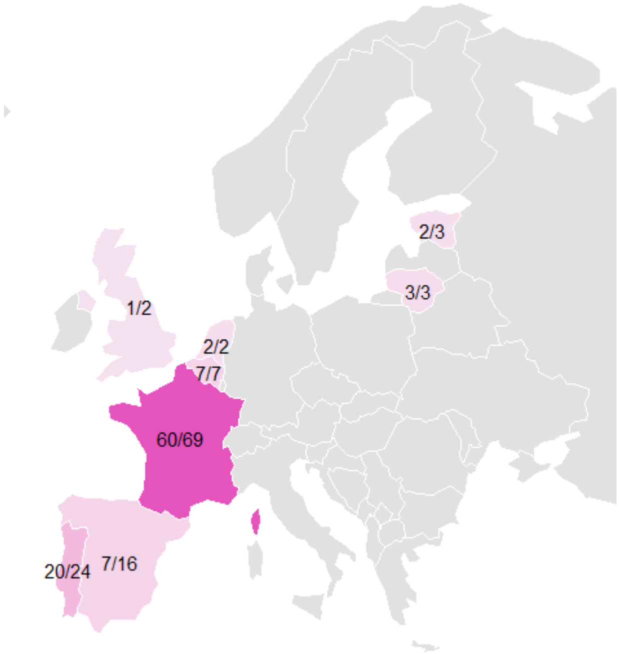

Supplementary Figure 2: Barplot of mutations in FFPE and plasma in pleural mesothelioma

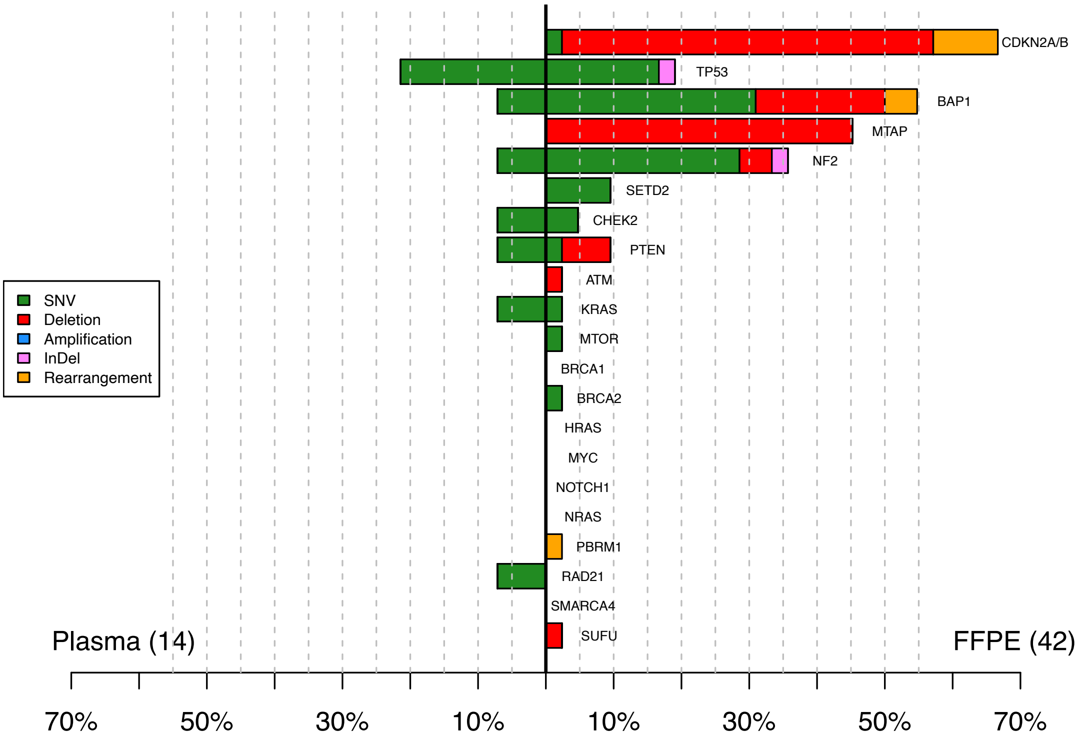

Supplementary Figure 3: Barplot of mutations in FFPE and plasma in thymic epithelioid tumors

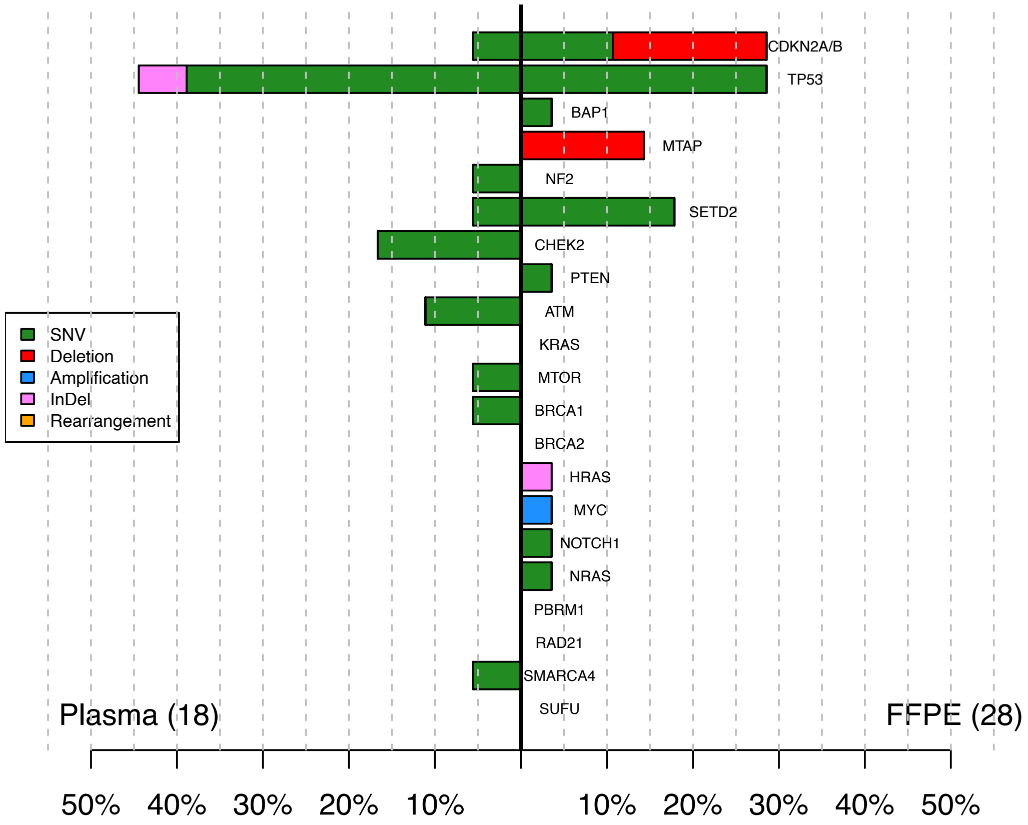

Supplementary Figure 4: Type of MTB treatment orientation

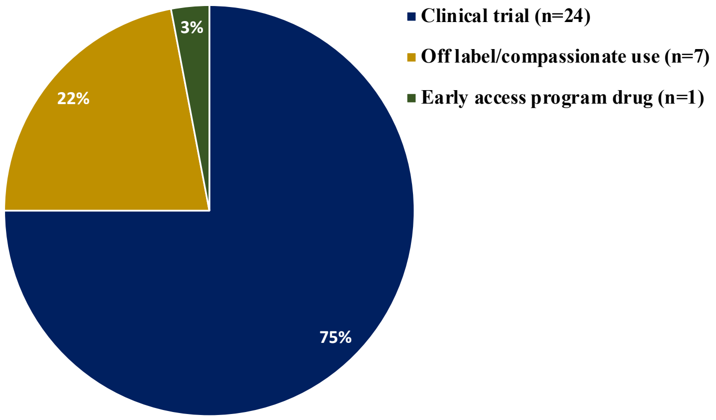

Supplementary Table 2: MTB treatment recommendations

MTB recommendation	n° patients
Investigational MAT2A inhibitor	**7**
Investigational p53-MDM2 inhibitor + CDK4/6 inhibitor	**5**
Investigational PARP inhibitor + immune checkpoint blockers	**4**
Immune checkpoint blockers	**4**
Investigational AKT inhibitor	**3**
mTOR inhibitor	**2**
KRAS G12C inhibitor	**1**
MEK inhibitor	**1**
Farnesyltransferase inhibitor	**1**
MET inhibitor	**1**
Investigational IGF-1R-targeted drug	**1**
Imatinib	**1**
FGFR inhibitor	**1**
**Total**	**32**
